# Supplementary material for: Are carbon emissions trading and green financial instruments synergistic? -Comprehensive quantitative research based on content analysis
Source: PLoS One. 2024 Mar 7;19(3):e0298601. doi: 10.1371/journal.pone.0298601 (PMC10919601; doi:10.1371/journal.pone.0298601)
Supplement: S1 Data — (ZIP) [file pone.0298601.s001.zip › Data/地级市Z_F工作报告.zip!/┴╔─■/░░╔╜╩╨/└┤╫╘╣1⁄2╓┌║┼í╛┬φ┐╦╩2╛▌═°í┐_2005.docx]

各位代表：

现在，我代表市人民政府向大会报告工作，请予审议，并请市政协各位委员及列席人员提出意见和建议。

一、二○○四年政府工作回顾

2004年，是鞍山振兴老工业基地实现良好开局的一年，是全市国民经济和社会事业加速发展并登上重要台阶的一年。过去的一年，在市委领导下，各级政府带领全市各族人民以邓小平理论和“三个代表”重要思想为指导，认真贯彻党的十六大和十六届三中、四中全会精神，按照市委九届七次、八次全会部署，全面落实科学发展观，认真组织重大专项推进，实施工业强市方略，较好地完成了市十三届人大二次会议通过《政府工作报告》所确定的各项任务，全市老工业基地振兴和全面建设小康社会取得新的成就。

――经济加速发展。初步核算全年实现地区生产总值突破1000亿元，达到1006亿元，按可比价格计算，比上年增长16．1％。人均地区生产总值超过3500美元，登上一个重要台阶。地区综合经济实力位居全国50强。

――综合财力明显增强。地区全口径财政一般预算收入达到105．2亿元，比上年增长43．1％，净增31．7亿元。地方财政一般预算收入达到40．2亿元，增长39．6％，净增11亿元。

――鞍钢生产经营再创历史新高。铁、钢、钢材产量分别达到1157万吨、1133万吨和1060万吨。实现产品销售收入470亿元，比上年增长49．3％。实缴税金55．6亿元，增长54．3％。为全市经济和社会发展做出重大贡献。综合竞争力跻身世界钢铁企业第八位。

――工业经济效益大幅度提高。全市规模以上工业企业实现销售收入839亿元，比上年增长34．8％。实现利润106亿元，增长2．9倍。工业经济综合效益指数达到218％，提高88．6个百分点，为历史最好水平。

――对外开放步伐加快。全年实际利用外商直接投资2．02亿美元（新口径），比上年增长38．2％。引进内联项目直接投资28亿元，增长25％。出口创汇10亿美元，增长1．4倍。设立鞍山公共保税仓库，填补了我市空白。

――城乡居民生活进一步改善。全年实现就业再就业12万人，城镇登记失业率为5．9％。城市居民人均可支配收入达到8262元，比上年增长11．1％。农民人均纯收入达到4253元，增长6．3％。这些成绩，标志着全市综合经济实力实现历史性跨越，增强了全市人民继续加速发展的信心和勇气。

一年来，我们主要做了以下八个方面工作：

（一）建设一个基地，培育三大产业，产业结构调整迈出新步伐。全年完成全社会固定资产投资217亿元，比上年增长23．6％。续建和新建投资亿元以上项目121个，竣工投产20个。其中，“一个基地、三大产业”项目83个，竣工投产8个。鞍钢冷轧2号线、厚板厂二期工程等重大项目相继竣工投产，西部500万吨精品板材基地完成土建和部分设备安装。海城纺织工业园成衣厂建成投产，20万吨差别化纤维一期工程开工建设。完达山年产10万吨液态奶项目竣工投产。成功举办了千山国际旅游节、第三届辽宁台湾周和海外学子创业周等重大招商活动，组织参加武汉、上海、广东等系列经贸洽谈活动，引进了台塑南亚等一批知名企业，欧文斯·科宁、台湾中橡等11家外商投资企业增资扩股。又有13个地方项目列入国家支持东北老工业基地调整改造第二批国债计划。全市省级高新技术企业达到155户，实现高新技术产品产值135亿元，增长31％。高新区新增入区项目199个，实现地区生产总值增长28％，地方财政一般预算收入增长19．9％。

（二）推进体制创新和机制创新，经济活力进一步增强。推广“两啤一化”模式，鞍山一工、北方铸钢和矿山机械等国有企业对外合资合作取得实质性进展。完成39户地方国有企业改制工作。343户符合条件的市属企业全部转属县（市）区。对53户企业依法实施破产。焦耐院、冶金院按照现代企业制度实施了分立式改制。制定出台了《关于加快非公有制经济发展的决定》，对非公有制纳税五十强和促进就业五十佳企业给予重奖。全年新办私营企业2027户，新登记个体工商户11665户。宝得公司、西洋集团、后英集团、衡业公司四家非公有制企业销售收入超过10亿元。宝得公司、西洋集团两家企业纳税均超亿元。西洋牌复合肥商标被认定为国家驰名商标。全年非公有制经济实现增加值478亿元，比上年增长15％。实缴税金21亿元，增长24．6％。

（三）大力支持鞍钢改革发展，地企合作迈出新步伐。坚持地方与鞍钢高层联席会议制度，在支持鞍钢西部新区建设、铁矿资源保护开发、推进地企合作等9个方面，明确了22项工作内容及具体目标。出台了《关于支持鞍钢主辅分离、辅业改制、分流安置富余人员的若干规定》。以2003年鞍钢上缴地方税收为基数，用新增财力的50％建立支持鞍钢发展专项资金，主要用于支持鞍钢重大项目建设的配套补助。促成鞍钢购并市第一轧钢厂和冷弯型钢厂等地企合作项目，冀东水泥一期工程竣工投产，1000万吨铁矿石采选项目开工建设。鞍钢血站划归地方管理，与鞍钢签订了10家医疗和医疗保险机构属地化管理移交协议，鞍钢剥离办社会职能取得新进展。

（四）坚持“一主三化”方针，认真解决“三农”问题，县域经济实现新发展。贯彻中央1号文件精神，全面落实“两减免、三补贴”政策，农民人均政策性增收56．80元。积极推进了农村第二轮土地延包工作。全市县级以上现代农业园区达到85个，市级以上重点龙头企业达到27家。全年粮食产量达到120万吨，为历史上第三个高产年。预计实现农业总产值97亿元，比上年增长16％。向县（市）区下放60项市级管理权限，取消市对县（市）区的税收分成，增强了县（市）区自主发展能力。海城、台安、岫岩和千山区经济总量占全市的比重达到44．7％。全年新建村镇住宅54万平方米、排水设施66公里，新增自来水用水人口5．9万人、集中供热面积23．6万平方米，建设农村公路748公里。积极发展少数民族地区经济，岫岩综合实力跃居省内8个少数民族自治县之首。启动农村信用社综合改革试点工作，超额完成了资本金募集任务。

（五）加快发展第三产业，城乡市场进一步繁荣。完成玉佛苑二期土建工程。“鞍钢工业之旅”被国家旅游局命名为“国家工农业旅游示范点”。全年接待国内旅游者690万人次，境外旅游者5．8万人次，实现旅游综合收入50亿元，比上年增长25％。积极发展新型流通业态，各类超市和连锁店发展到490家。社区服务网点发展到11638个，从业人员2．9万人。全年实现社会消费品零售总额195亿元，增长13．8％。实现第三产业增加值360亿元，增长13．3％。严厉打击非法传销活动，严肃查处食品、药品、农资等领域的制假售假行为，营造了良好的市场秩序和消费环境。

（六）全面加强软硬环境建设，经济发展环境不断优化。启动了站前景子街改造、南沙河综合整治、引汤入鞍二期工程、节能环保集中供热等一批重大基础设施建设项目。完成市客运站改扩建工程。完成兴盛路南延、千山西路改造、玉佛山隧道南洞扩建等道路建设。改造棚户区13万平方米，新建城市住宅98万平方米。改造小街小巷23条、居民小区56个，新增城市绿地80万平方米。完成大孤山供水和城区3条排水明沟改暗渠工程。取缔4吨以下燃煤锅炉173台，新增集中供热面积112万平方米。城区全年空气质量二级以上天数达到256天，比上年增加30天。对南沙河、海城河、五道河流域90户污染企业进行了集中整治，完成7项水污染治理项目。海城生态示范区通过国家验收。国家环保模范城市28项考核指标有22项达标。

软环境建设又有新进展。贯彻《行政许可法》，制定出台了进一步优化经济发展环境的意见，废止行政许可150项，取消48个行政事业单位的执法主体资格。将符合条件的201项行政许可全部纳入市公共行政服务中心办理。以建设政府、企业、个人三大信用体系为目标，全面启动了“诚信鞍山”建设工程。

（七）切实解决民本民生问题，就业和社会保障工作取得新成果。探索总结出“一方为主、多方联动、重点突破、整体推进”的“1＋X”就业模式。建成市创业服务中心二期工程，被国家劳动和社会保障部确定为全国首批“创办和改善你的企业”试点城市。整合职业技术教育资源，组建了鞍山技师学院。完成职业技能培训7万人，培训就业率达到60％。养老保险实现了市级统筹。城镇居民最低生活保障标准提高到205元，农村最低生活保障标准提高到42．5元。及时足额发放养老保险金11．3亿元，比上年增长8％；发放失业保险金2．3亿元，增长18％；发放城乡最低生活保障金8508万元，增长14％；失业并轨人员内欠累计偿还率达97．7％。对城乡10万户困难群众实施了临时救助，为贫困户建房325户，资助贫困家庭学生就学3．3万人。20个省定贫困村人均增收284元。认真开展矛盾纠纷调处工作，受理群众来信来访4．1万件次，受理市民投诉2．3万件次。人大代表建议和政协委员提案办复率达100％。台安被省政府命名为“无毒县”。建立社区、巡防、阵控三大警务体系，抢劫、抢夺和入室盗窃发案率明显下降，破案率明显上升，“平安钢都”建设取得明显成效。

（八）统筹经济和社会发展，社会事业取得全面进步。开工建设一中新校园，新建扩建了岫岩高中和台安高中，高中扩招13．5％。鞍山师范学院通过国家教育部评估验收。对文化市场和出版物市场进行专项治理，组织开展了丰富多彩的群众文化活动，喜获省艺术节金奖五连冠。制定突发公共卫生和安全事件应急条例暂行规定，建成市传染病医院呼吸道传染病房，完成医疗紧急救援中心主体工程。全面加强农村和社区医疗卫生工作，台安县新型农村合作医疗试点工作得到省政府充分肯定。开展党政机关帮建社区活动，86％社区“一站四室”达到省规定标准。深入开展国防教育，巩固了双拥模范城创建成果。成功举办了市九运会和首届人口文化节。城区34万有线电视用户接入宽带网。全市青年志愿者、巾帼志愿者和老年志愿者队伍不断壮大。

各位代表，在振兴老工业基地起步之年，我们取得了比年初预想要好的发展成果，这是省委、省政府和市委正确领导及市人大、政协监督支持的结果，是全市各族人民团结拼搏、勤奋工作的结果，也是社会各界大力支持、热心帮助的结果。在此，我代表市人民政府全体成员，向辛勤工作在各条战线和各个岗位上的全体劳动者，向为鞍山经济和社会发展做出重大贡献的鞍钢等中省直单位，向驻鞍部队和武警官兵，向给予政府工作大力支持的人大代表、政协委员、各民主党派、工商联、各人民团体和社会各界人士，以及国内外所有帮助支持鞍山建设和发展的朋友们，致以崇高的敬意和衷心的感谢！

回顾过去一年，我们也清醒地看到，在前进的道路上还有许多困难和问题，政府工作也存在着不少缺点和不足，人民群众还有一些不满意的地方。主要表现在，经济体制和运行机制方面的矛盾仍很突出；产业结构调整任务还很繁重；经济增长方式粗放，整体竞争力不强，对外开放水平不高；社会保障能力脆弱，就业再就业压力还很大，部分城乡群众生活还很困难，影响社会稳定的因素仍然存在；经济发展环境仍不够宽松，与先进地区相比差距仍然较大；一些政府工作人员服务意识不强，依法行政水平不高，仍然存在形式主义、官僚主义作风，甚至还有贪污腐败行为。上述矛盾和问题，我们将高度重视，认真解决。

各位代表，振兴老工业基地任重道远，我们将以全新的风貌、顽强的斗志迎接新的挑战，决不辜负全市人民的期望。

二、二○○五年主要任务

2005年是“十五”计划最后一年，也是振兴老工业基地的关键之年。按照市委九届七次、八次和九次全会做出的部署，新的一年，政府工作的总体思路是，以邓小平理论和“三个代表”重要思想为指导，深入贯彻党的十六大和十六届三中、四中全会精神，坚持以人为本执政理念，以科学发展观为统领，以振兴老工业基地为中心，以改革开放和科技进步为动力，认真实施工业强市方略，加快推进体制创新和机制创新，加快推进产业产品结构调整，加快推进经济增长方式转变，全面提升城市综合竞争力，努力构建和谐鞍山，促进全市经济和社会发展在高起点上实现新跨越，在辽宁建设国家新型产业基地和成为新的重要增长区域中发挥主力军作用。

2005年全市经济与社会发展的主要预期目标是：

――继续提升综合经济实力。努力使国民经济和社会事业保持较快增长，实现国内位次前移。全年预期实现地区生产总值比上年增长13％以上。财政一般预算收入增长14％以上。全社会固定资产投资增长20％以上。社会消费品零售总额增长13％以上。

――继续提高核心竞争力。以建设“一个基地，三大产业”为重点，规划实施投资亿元以上项目达到200个。其中，续建项目100个，新开工项目48个。当年竣工30个。基本构筑起以工业为主体，高新技术产业为先导，现代农业为基础，服务业全面发展，能够支撑鞍山全面振兴的产业构架。

――继续扩大对外吸引力。打造东北地区一流的投资环境，让更多的人才选择鞍山，更多的资本涌入鞍山，更多的企业落户鞍山，更多的本地非公有制企业茁壮成长。实际利用外商直接投资增长30％以上。引进内联资金增长40％以上。

――继续激发地区经济活力。年内完成国有资本从一般竞争性领域全部退出，新注册各类非公有制企业3000家，新增个体工商户15000户。基本形成国有经济以鞍钢为主，地方经济以非公有制为主的发展格局。

――继续增强城市凝聚力。推进先进文化城市建设，开展全国文明城市、国际健康城市、国家环保模范城市和双拥模范城创建活动，构建三个文明协调发展的和谐社会，让老工业基地振兴成果惠及更广大群众。全年完成就业9万人。城市居民人均可支配收入增长10％，农民人均纯收入增长6％。

按照上述指导思想和主要目标，新的一年，我们将重点做好以下十个方面工作：

（一）推进“一个基地、三大产业”项目建设，继续优化产业结构

做大做强精品钢材基地。重点推进18个项目，当年完成投资65亿元。积极支持鞍钢完善东部1100万吨钢配套工程和西部500万吨精品板材基地等11个续建项目。帮助鞍钢建设总公司等改制分流企业实现平稳过渡。协助鞍钢列入国家厂办集体企业改制试点名单，制定出台相关配套政策，引导鞍钢集体企业走向市场。支持鞍钢盘活三车厂闲置资产，支持冀东水泥二期工程建设。引导地方企业发展钢材精深加工，拉长钢铁产业链条。支持中油天宝集团30万吨大直缝石油焊管、西洋集团400万吨热轧板及100万吨冷轧板、峰驰集团50万吨冷轧板等项目建设。

全面提升三大支柱产业。装备制造业，支持鞍钢机械制造公司做大做强，推进海诺集团二期工程等5个续建项目，开工建设衡业集团改装汽车和载重汽车生产线等11个项目。轻纺工业，重点推进海城纺织工业园项目建设，促进味邦制药60万支胶原酶等27个项目开复工，建成后英集团大豆深加工项目。矿产品加工业，按照资源有序利用和可持续发展原则，搞好铁、菱镁、滑石等矿产品精深加工，淘汰工艺落后、污染严重的粗加工项目。支持岫岩菱镁工业园等11个项目建设，建成1000万吨铁矿石采选等5个项目。同时，推进博达化工等10个工业项目和17个国债项目建设。争取无油空压机等项目列入国债专项。完成鞍山炼油厂转产项目前期工作。

组织开展工业升级年活动。调整工业区域布局，推进城区工业企业向园区搬迁改造。制定首批60户企业搬迁规划和相关配套政策，5户以上企业在年内实现搬迁改造。实施名牌战略，引导企业大力发展专、特、精终端消费产品，新增两个国家驰名商标。制定电力紧张应急方案，引导企业合理避峰用电。制定鞍山产业结构调整指导目录，鼓励企业推行清洁生产，发展循环经济。

（二）高度重视“三农”问题，统筹城乡协调发展

全面深化农村改革。及时足额发放三项补贴资金，全面取消农业税。实行严格的耕地保护制度，依法稳定农村土地承包关系。完善农村税费改革配套措施，开展村级财务会计委托代理试点，研究化解乡村两级债务的有效途径和办法，坚决遏制新的债务发生。加大财政资金对农业的投入，建立工业反哺农业、城市支援农村新机制。新建10个无公害农产品质量检测站，培育10个以上带动力强的农村合作经济组织，提高农民进入市场的组织化程度。

积极调整农业产业结构。制定出台农业产业化发展规划及相关配套政策，建立农业产业化发展专项资金。大力发展绿色无公害农产品，以优质稻米、鲜细菜、高油大豆、南果梨、食用菌、肉用牛羊、肉蛋鸡等特色产品为主，培育壮大32个农业产业化基地。推广“公司＋基地＋合作经济组织＋农户”模式，订单农业达到120万亩。培育规模以上龙头企业100家，现代农业园区100个。实施天然林保护工程，新增封山育林10万亩，植树造林8万亩。加快发展畜牧业，新建10个标准化畜牧养殖小区。全年农业总产值突破100亿元。

大力推进农村工业化。优化县域工业布局，实施亿元以上工业项目76个，当年完成投资75亿元。围绕发展农副产品精深加工，重点支持龙兴果蔬功能饮品、永安滑子蘑深加工等20个亿元以上项目建设。鼓励支持农民向小城镇集中和从事二、三产业，完成农村劳动力转移2万人。全年非农产业主要指标增长15％以上。

加强农村水利和农田基本建设。以东部山区和西部沿河为重点，实施农村饮水、抗旱、蓄水、排涝200项骨干工程，整治河道200公里，治理水土流失面积14万亩，建设优质粮标准化农田20万亩。启动全程农业机械化工程，引进各类农机具1500台（套）。

（三）加快发展第三产业，全面提高现代服务业水平

全面提升旅游业竞争力。制定出台《鞍山市旅游发展总体规划》。按照经贸、旅游、会展“三位一体”思路，积极争办各类展销、洽谈、论坛活动，办好第二届宝玉石博览会。启动千山中南部景区改造工程和温泉度假区滨河景观工程。全年实现旅游综合收入增长15％以上。

大力发展现代服务业。推进二十一世纪置业广场、站前景子街、中海物流中心等大型商业物流设施建设，建成大德家居超市和大福源超市。引进国内外知名企业改造盘活银座大厦、供销城、北辰大厦、亚光大厦、储运公司等商业设施。整合农村、社区商业网点，大型超市和连锁店超过500家。培育投资咨询、市场调查、职业介绍等各类中介组织，完善产权交易、技术转让等各类要素市场。

全面加强城乡市场监管。建立农产品质量安全体系，推行农副产品市场准入制度，加强食品、药品市场监管，营造安全放心的消费环境。整顿规范市场价格秩序，明码标价率达到90％以上。制定并实施畜禽屠宰管理条例和报废汽车回收管理办法，依法加强特殊商品交易市场管理和行业专项整治。

（四）大力实施科技兴市和人才强市战略，加快推进经济增长方式转变

全面实施科技兴市战略。支持宝得公司等40户企业开展技术创新。继续办好海外学子创业周活动，建立10个稳固的海外学子联系渠道，海外学子库人数超过240人。选择10个以上重大项目开展国际科技合作。扩大院校合作成果，重点联系高校达到100家，以高校为技术依托的企业达到150家。加快科技创新体系建设，重点规范24个研发中心和中试基地。深入开展“科技兴农”和“科技兴企”活动，引进推广重大科研成果65项。

加快发展高新技术产业。建设中国北方冶金自动化产业基地、金融设备产业园。启动信息化改造“双百”工程，支持三和轧钢、吉兆电子、华冶动力等20户省示范企业信息化改造。支持中冶焦耐和中冶北方工程技术公司做大做强。省级高新技术企业达到165户，高新技术产品产值达到145亿元。

全面提升高新区建设管理水平。完善高新区基础设施，优化投资发展环境。建成南亚实德塑料管材生产线，完成海诺集团超大型混凝土搅拌站产业化、电子电力公司光纤测控系统产业化基地土建工程，开工建设凯信环保矿山设备、华冶特种变压器等项目。高新区全年实现地区生产总值比上年增长30％以上，财政收入增长20％以上。

实施人才强市战略。推进振兴老工业基地人才工作行动计划。选拔和培养学科带头人50名，新建博士后工作站和博士后科研基地3个。引进全日制本科以上人才超过2000人。推荐50名非公有制企业经营者进大学深造。引导大中专毕业生向非公有制企业和农村流动，新增农村实用人才5000人。

（五）全面深化各项改革，加快体制创新和机制创新

深化国有企业改革。重点推进10个重大重组购并项目，年内完成一般竞争性领域最后87户国有企业改制任务。其中，对17户市属工业企业通过国有股转让、资产重组、依法破产等多种方式改制退出。对已转属的70户国有企业，加大指导力度，确保完成规范化改制。深化粮食购销体制改革，对粮食企业进行公司制改造，建立完备的现代企业制度。

大力发展非公有制经济。落实促进非公有制经济发展的各项政策，合理使用非公有制经济发展专项资金，支持宝得公司、西洋集团、后英集团、衡业公司等一批非公有制骨干企业做大做强，对外向型、就业型、科技型、农产品加工型、社区服务型五类小企业给予扶持，对列入国家和省专项计划的非公有制企业项目给予配套支持。全年非公有制经济主要指标比上年增长15％以上。

推进市政公用事业改革。按照政企分开、政事分开、建管分开原则，加快推进城市供水、供气、供热、公共交通、污水和垃圾处理等市政公用事业产权制度改革，建立规范的法人治理结构。对自来水公司等公益服务类企业，引进域外资金，积极慎重地实行国家控股或参股的产权制度改革。制定出台特许经营管理办法，在推进市政公用事业市场化进程中，强化政府的监控职能。

积极实施事业单位改革。对20家行政委托类事业单位，实行政事分开，重新界定职能，核定人员编制，规范经费管理。对社会公益类事业单位，剥离经营实体和后勤服务机构，强化公益服务职能。深度推进经营服务类事业单位转企改制，使其成为法人实体和市场竞争主体。

（六）实施“一开三促”发展战略，全面扩大对内对外开放

强化招商引资工作。鼓励外商投资基础设施和基础产业，推进金融保险、商业流通、旅游服务等领域利用外资工作，全年推出投资500万美元以上重点招商项目120个。以台湾、日韩、香港、欧美为重点区域，以国内外知名大企业为招商对象，办好香港（鞍山）投资推介会。积极推进以外引外和以商招商，促进外商投资企业增资扩股。强化签约项目的跟踪落实，提高外资到位率。

积极推进对外经贸工作。制定出口鼓励措施，扩大钢材、轻纺、机电和高新技术产品出口。扶持鞍钢国贸等21家出口骨干企业发展，推进海天服装等15家企业由出口供货向直接出口转变，扩大10户骨干三资企业出口，吸引省内外公司和个人来鞍设立外贸出口企业。挖掘友好城市和友好关系城市资源，积极开展对外交流与合作，争取国际多边和双边援助项目。新批2家外经窗口公司，实现3户企业境外投资。

加强区域经济合作。制定参与辽宁中部城市群和大连东北亚国际航运中心的区域经济合作规划，找准区域定位，促进产业对接，实现优势互补。坚持“重外不轻内、重大不轻小”原则，以长三角、珠三角为重点招商区域，积极吸引国内知名大企业来鞍投资。

（七）加强城乡建设与管理，改善人居环境和生态环境

认真做好城乡建设规划编制工作。完成城市总体规划调整大纲，编制新一轮土地利用总体规划，全面制定城市分区控制性详细规划。完善矿山土地复垦和植被恢复总体规划，制定千山风景区和玉佛山风景区保护性详细规划。严格执行土地供应年度计划，严厉查处各类违规建设行为，维护规划的权威性。

加强城乡基础设施建设。推进引汤入鞍扩建、城市集中供热工程建设，完成第二污水处理厂二期主体工程。开工建设深营路南段及隧道工程，形成湖南地区至高新区和千山风景区的快捷通道。规划改造二一九公园和玉佛山风景区道路，研究解决永乐公园被侵占问题，切实提高管理水平，为市民提供更多、更好的休闲娱乐场所。新建曙光路沙河大桥，扩建玉佛山隧道北洞，完成南环线、曙光路北段等道路大修改造。建成医疗垃圾无害化处理中心，改造后峪、杨柳河支线的排水明渠及部分陈旧供水管网。改造中省直单位所属居民小区250万平方米，绿化、美化50个居民小区，创建10个智能小区。完成深营路等24条道路行道树栽植、补植，新建城市基础绿地10万平方米。完成棚户区改造任务，市区新建住宅95万平方米。

推进城乡环境综合整治。深入开展国家环保模范城市创建活动，确保28项考核指标有24项达标。实施12项重点环境污染治理工程，取缔4吨以下供暖燃煤锅炉110台，城市空气质量二级以上天数达到275天。治理南沙河选矿企业废水、千山旅游度假区洗浴和餐饮污水超标排放问题。继续推进南沙河综合整治，建设立山段防洪堤及两道橡胶坝，具备截流条件。做好杨柳河综合整治前期工作。清理整顿露天烧烤、占道刷车和马路市场。研究制定城乡结合部和城市主要出口综合改造规划，整顿城区广告牌匾及“四乱”广告，全面改善市容市貌。

加强资源保护和合理开发。建立地下水动态观测网和水污染防治网，合理开发利用地下水资源和地热资源。全部查封城市无证自备井，有计划关闭有证自备井。做好土地开发整理和矿山复垦工作，完成城市周边矿山生态恢复100公顷。整顿矿产资源开发秩序，加强矿业权市场建设，坚决制止滥采乱挖和资源浪费现象。

加强小城镇规划与建设。制定鞍山市中心镇建设管理办法，调整西柳、高力房、偏岭等小城镇建设总体规划，编制完成牌楼等10项小城镇建设详细规划。新建村镇住宅35万平方米，修筑道路200公里，新增公共绿地10公顷、集中供热20万平方米。全部完成农村防氟改水工程，新增自来水受益人口3万人。

（八）认真落实国家调控政策，切实做好财税金融工作

强化税源建设和税收征管。用好支持企业发展的各类专项资金，促进产业升级，壮大财源税源。加强土地出让金征缴，整合政府财力资源，推进非税收入收缴制度改革，增加可支配财力。在城区实行属地税收征管体制，理顺市与县（市）区两级财政分配关系，规范县乡财政体制。依法打击偷税、逃税、骗税、抗税等违法行为，确保应收尽收，及时入库。

调整和优化财政支出结构。推进部门预算编制改革，强化支出预算的刚性约束。扩大政府采购范围和规模，提高财政资金使用效益。推进国库集中收付制度改革，建立绩效评价机制和理财过错追究办法。规范财政供养范围，依法加大农业、科技、教育、卫生等事业支出，保证工资发放、社保支出、党政机关正常运转资金需要。建立政府债务预警机制，提高风险防范能力。

引导和改善金融服务。建立健全信贷担保体系，引导金融机构加大对“一个基地、三大产业”建设项目、农业开发项目、再就业小额贷款的资金投入。进一步加强与国家开发银行的合作，积极争取各类政策性贷款。全面推进农村信用社改革试点，构建农村新型金融服务体系。积极推进市商业银行综合改革，完善法人治理结构和经营管理机制。大力发展资本市场，引导企业上市融资。严厉打击金融欺诈和逃废债行为，防范和化解金融风险。

做好“十一五”规划编制工作。高质量完成首次经济普查任务，为规划编制提供准确的基础数据。按照宏观性、战略性、政策性和指导性原则，建立咨询审议制度和民主化编制程序，编制出一个能够把鞍山带入全面小康社会的中长期发展规划。

（九）强化就业和社会保障工作，继续改善人民生活

千方百计扩大社会就业。实施就业优先战略，统筹城乡就业，深化和推广“1＋X”就业模式，城镇登记失业率控制在6％以内。拓展创业服务中心功能，全年新办再创业企业达到1万个。调整城市最低工资标准，由每月290元提高到400元。以鞍山技师学院为龙头，整合职业教育资源，加强技能培训，全年培训就业率达到70％。实施异地就业战略，积极引导失业人员和农民外出打工，全年劳务输出10万人。落实再就业政策，建立就业备案制度、就业准入制度和实名就业制度，就业稳定率达到60％以上。加强劳动稽查，依法规范企业用工行为。成立农民工服务中心，维护打工农民的合法权益。

规范和完善社会保障体系。制定社会保险扩面征缴实施细则，以非公有制企业为重点，依法扩大社会保险覆盖面。强化社会保险费征缴，保证养老金和失业金及时足额发放。制定破产企业退休人员医疗参保政策和困难企业职工大病医疗救助办法，保障困难群体的基本医疗需求。

认真做好扶贫帮困工作。全面推行城乡居民最低生活保障制度，将符合条件的城乡困难居民全部纳入保障范围，实施动态管理。以建立社会救助长效机制为目标，健全困难群体数据库，完善社会捐助制度，构筑规范化的城乡救助体系，重点关注弱势群体特别是残疾人生产生活问题。深入推进开发式扶贫，重点帮扶15个省定贫困村脱贫致富。

（十）加速构建和谐鞍山，努力保持社会稳定

大力发展社会各项事业。围绕三年争创全国文明城市目标，积极开展群众性精神文明建设活动。以贯彻落实《公民道德建设实施纲要》为重点，在全市范围内组织开展诚实守信教育活动，进一步加强未成年人思想道德教育。搞好师德师风建设，着力解决教育热点问题，严格控制农村中小学辍学率，在省内率先实现学校D级险危房为零的目标，全面推进“双高普九”工作。加快教育现代化建设，推进“数字化校园”工程，完成城市中小学多媒体教室建设，实现农村初中以上“校校通”。加强高等院校建设。完成新一中和海城高中建设工程，确保秋季招生。深化文化体制改革，大力繁荣文化事业，加强城乡公共文化基础设施建设，发展具有鞍山特色的文化产业。开展国际健康城市创建活动，完善疾病预防控制、突发公共卫生事件医疗救治和疫情信息网络，健全公共卫生体系。编制农村卫生服务体系发展规划，继续搞好新型农村合作医疗试点。深入开展全民普法教育，深度推进依法治市。开展全民健身活动，提高竞技体育水平，备战省十运会。深入开展国防教育，支持驻军后勤保障社会化。加强人口问题的战略研究和预测。

全面加强社区建设。社区“一站四室”全部达到省规定标准。加强社区劳动保障平台规范化建设，实现市、县（市）区、街道、社区四级联网。高标准、高质量完成社区居委会换届选举。在城区老社区全面推行开放式物业管理，创建全国社区建设示范市。

认真解决社会热点难点问题。贯彻信访条例，建立大信访工作机制和重大事件及异常突发事件责任追究制度，全面加强接待群众来信来访和受理市民投诉工作。认真搞好矛盾纠纷排查调处，对信访案件实施源头治理。落实信访分级管理责任制，着力解决土地征收、房屋拆迁、企业改制、劳资纠纷、公益服务等方面问题，维护人民群众的切身利益。

加强安全生产和社会治安综合治理。贯彻实施《安全生产许可证条例》和《特种设备安全监察条例》，对高危行业实行严格的安全生产许可证制度。健全安全生产监督管理体制，排查和整改安全隐患，防止重特大安全事故发生。巩固提升畅通工程国家A类一等模范城市创建成果，积极争创国家畅通工程绿色交通示范城市。加强社会治安防控体系建设，构筑打防控一体化警务格局，提高防范打击各种犯罪活动和处置各类突发事件的能力。开展平安社区创建活动，打造平安钢都，争创安全城市。

三、推进政府职能转变，加强行政能力建设

贯彻落实《行政许可法》和国务院《全面推进依法行政实施纲要》，切实转变政府职能，加快建设服务政府、法治政府、责任政府，不断提高政府行政能力。

（一）全面提高机关行政效能，努力建设服务政府

完善公共行政服务体系。按照《行政许可法》要求，继续清理行政审批事项，逐一规范办理时限、申报材料、收费标准和勘察内容，在省内做到审批环节最少和审批时限最短。对各部门依法予以保留的行政许可项目，归口为一个处室（窗口）统一管理，实施相对集中许可权制度。健全公共行政服务信息网络，推广一站式并联审批。落实行政监察、定点监控、暗访回访等制度，提高公共行政服务水平。

建立政府信息公开制度。落实《鞍山市政府信息公开暂行办法》，对政府管理规章制度、经济社会发展规划、基础设施建设规划及方案、公共资金使用情况以及其他与人民群众利益密切相关的行政事项，利用政府公报、政府网站和新闻媒体，及时向社会公开，将政府工作自觉置于人民群众的监督之下。拓展党政信息网功能，加快建设电子政务。

打造诚信鞍山。落实政府、企业、个人三大诚信体系建设方案，搭建覆盖全市的信用信息基础平台。强化行政责任追究，严厉惩处政府部门及工作人员失信违约行为。完善企业信用联合征信机制和企业信用评价体系。以重点人群为对象，探索建立个人信用档案，开展个人信用评价评估业务。

（二）推进法制建设和依法行政，努力建设法治政府

发展社会主义民主。贯彻落实市委人大工作会议和政协工作会议精神，自觉接受人大依法监督和政协民主监督。完善向人大及其常委会报告工作制度和主动与政协协商制度，落实重大事项提前报告制度，拓宽工作联系渠道，保证人大代表、政协委员的知情权和监督权。落实人大及其常委会的各项决议，认真办理人大代表建议和政协委员提案。支持工会、共青团、妇联等社会团体工作，保障人民群众当家作主权力。

完善民主决策机制。健全重大事项社会公示制度、听证制度和专家咨询制度，建立多种形式的决策咨询机制和信息支持系统。对专业性、技术性较强的重大事项，认真组织专家论证、技术咨询和决策评估，扩大人民群众的参与度。

强化行政立法工作。坚持以人为本理念和法制统一原则，科学选定行政立法课题。健全和完善行政立法程序。对涉及群众切身利益或对改革、发展、稳定有重大影响的立法项目，实行“开门立法”，充分听取社会各界和人民群众的意见和建议，不断提高行政立法质量。

加强政府制度建设。严格执行政府工作规则，建立决策跟踪反馈和责任追究制度，强化政府层级监督，完善行政执法责任制和评议考核制。认真开展行政复议工作，健全执法监督体系，实现执法重心下移，保证依法行政的有效性。

积极推进行政综合执法。按照政策制定职能与监督处罚职能相对分开、监督处罚职能与技术检验职能相对分开的原则，科学界定行政执法机构和职能，先行在城市管理领域进行试点，逐步向其他领域拓展，尽快建立起权责明确、行为规范、监督有效、保障有力的行政执法体制。

（三）规范行政职责和行为，努力建设责任政府

进一步理清部门职责。坚持职权、义务、责任三结合原则，科学界定政府部门职责分工，从制度上根治相互争权、遇事推诿、办事拖拉的体制弊端。重点解决城乡规划、土地审批、安全生产、公共卫生、市场监管、交通管理、小区建设等方面存在的职能交叉和职责不清问题，坚决杜绝错位、缺位和越位现象发生，形成各司其职、权责统一的行政管理体制。

严格追究行政过错行为。严厉查处行政机关特别是领导干部滥用职权、玩忽职守、徇私舞弊等违法违规行为。对食品卫生、安全生产、公共卫生等方面的失察、失职、渎职行为，严格进行责任追究。全面推行绩效量化考核，着力解决机关中存在的不作为和乱作为问题。

切实加强政风建设。弘扬求真务实精神，坚决克服虚报浮夸、滥用民力、强迫命令等形式主义、官僚主义作风。精简会议和文件，清理各类检查、评比、考核活动，减轻基层和企业负担。认真纠正部门和行业不正之风，构建教育、制度、监督并重的预防和惩治体系，树立清正廉洁的机关形象。

各位代表，全面振兴老工业基地我们已经迈出了可喜的一步。站在历史发展的新起点上，让我们高举邓小平理论和“三个代表”重要思想伟大旗帜，万众一心，与时俱进，抓住机遇，乘势而上，为谱写老工业基地振兴新篇章而努力奋斗！
